# Why can *Mikania micrantha* cover trees quickly during invasion?

**DOI:** 10.1186/s12870-024-05210-5

**Published:** 2024-06-07

**Authors:** Lihua Chen, Minling Cai, Qilei Zhang, Yanru Pan, Manting Chen, Xiaowen Zhang, Jirong Wu, Haoshen Luo, Changlian Peng

**Affiliations:** 1 Guangzhou Key Laboratory of Subtropical Biodiversity and Biomonitoring, Guangdong Provincial Key Laboratory of Biotechnology for Plant Development, School of Life Sciences, South China Normal University, Guangzhou, 510631 China; 2 School of Life Sciences, Huizhou University, Huizhou, 516007 China; 3 Research Institute of Tropical Forestry, Chinese Academy of Forestry, Guangzhou, 510520 China; 4 College of Life Sciences, South China Agricultural University, Guangzhou, 510642 China

**Keywords:** Sugars, Plant hormone, Branches, Tree canopy, Biological invasion, *Mikania micrantha*

## Abstract

The invasion of *Mikania micrantha* by climbing and covering trees has rapidly caused the death of many shrubs and trees, seriously endangering forest biodiversity. In this study, *M. micrantha* seedlings were planted together with local tree species (*Cryptocarya concinna*) to simulate the process of *M. micrantha* climbing under the forest. We found that the upper part of the *M. micrantha* stem lost its support after climbing to the top of the tree, grew in a turning and creeping manner, and then grew branches rapidly to cover the tree canopy. Then, we simulated the branching process through turning treatment. We found that a large number of branches had been formed near the turning part of the *M. micrantha* stem (TP). Compared with the upper part of the main stem (UP), the contents of plant hormones (auxin, cytokinin, gibberellin), soluble sugars (sucrose, glucose, fructose) and trehalose-6-phosphate (T6P) were significantly accumulated at TP. Further combining the transcriptome data of different parts of the main stem under erect or turning treatment, a hypothetical regulation model to illustrate how *M. micrantha* can quickly cover trees was proposed based on the regulation of sugars and hormones on plant branching; that is, the lack of support after ascending the top of the tree led to turning growth of the main stem, and the enhancement of sugars and T6P levels in the TP may first drive the release of nearby dormant buds. Plant hormone accumulation may regulate the entrance of buds into sustained growth and maintain the elongation of branches together with sugars to successfully covering trees.

**Supplementary Information:**

The online version contains supplementary material available at 10.1186/s12870-024-05210-5.

## Introduction

Shoot branching is crucial for plant development and determines plant architecture. Plant branches mostly developed from axillary buds formed in the leaf axil. Usually, the buds are in an arrested or very slow growing state under apical dominance and are known as dormant buds. Auxin has long been regarded as a key mediator of apical dominance and operates through two nonmutually exclusive models. One model is the auxin transport canalization-based model, in which auxin efflux from dormant buds is competitively inhibited by polar auxin transport (PAT) in the main stem to restrict bud growth, and hormones such as cytokinin (CTK) can activate bud outgrowth by regulating the accumulation of auxin efflux carrier PINs to promote auxin export [1, 2]. Another model is the second messenger theory model, in which auxin regulates the biosynthesis of other hormones, such as CTK and gibberellin (GA), which can enter buds to promote bud outgrowth [3–5]. However, studies of tall pea plants showed that the release of dormant buds is not associated with auxin but strongly correlates with sugar availability after decapitation. They proposed that sugar plays a dominant role in the early release stage of dormant buds, while auxin is prominent in the later stages of bud sustained growth [6, 7]. Recent studies have also focused on the regulatory mechanism of sugar in the outgrowth of dormant buds, including the nutritional role of sugar and the signalling role of the sucrose-T6P nexus model [8–10].

*M. micrantha* is a perennial climbing herb belonging to the genus *Eupatorium* in the Compositae family. It is native to Central and South America and has become one of the most harmful weeds in tropical and subtropical regions of the world [11]. It can reproduce by both sexual and asexual reproduction [12], and it exhibits rapid diffusion and spread. *M. micrantha* grows easily in forest margins, orchards, and riverbanks, along highways and railways, in low-lying wet open areas and in green areas of cities and towns. Natural secondary forests, plantations, shrubs, economic forests and scenic forests, especially those with low canopy density and scenic forests, are most harmed by *M. micrantha* [13]. Forests account for 80% of Earth’s plant biomass and 75% of the gross primary productivity of the Earth’s biosphere [14]. Reductions in forest area cause ecological and environmental issues and result in alterations of forest stand structure [15] and biodiversity losses [16].

*M. micrantha* is characterized by climbing trees and growing a dense ‘sunshade’ in the canopy, resulting in the covered tree being unable to carry out normal photosynthesis and eventually asphyxiating and dying [17]. In the wild, it was found that the sustained growth of the *M. micrantha* main stem without support after reaching the top of the canopy led to turning and creeping growth and ultimately covered the tree by growing many branches to wind and climb. A study has shown that rapid stem growth is the most significant feature of *M. micrantha*, with a maximum mean growth rate of 20 cm day ^− 1^ [18]. It can quickly produce new branches for vegetative propagation with a high ability to branch [12, 19]. The vigorous growth habits and reproductive capacity of *M. micrantha* stems are important reasons for climbing and covering trees quickly and successfully invading forests. However, the regulatory mechanism of how *M. micrantha* regulates its mass branching to cover trees is still unknown.

In this study, we simulated the covering process by producing a large number of branches after *M. micrantha* climbed to the tree canopy by a turning experiment, combining physiological changes in different parts of the main stem with transcriptome data of the starch and sucrose metabolism pathway and plant hormone signal transduction pathway, to explain the strategies of *M. micrantha* for covering trees by a hypothesis model, that is, the loss of support caused its main stem to be turning growth. The accumulation of sugars and T6P at the turning part of the main stem (TP) may first activate the release of dormant buds near the TP, and the accumulation of plant hormones further promotes buds to enter a sustained growth stage, ultimately maintaining rapid branch growth together with sugars.

## Materials and methods

### Plant materials and treatments

To simulate the phenomenon of climbing under the forest, *M. micrantha* seedlings were propagated through seeds collected from South China Botanical Garden, Guangzhou, China, and seedlings of approximately 3 cm in height were planted with native dominant trees (*C. concinna*). Trees of approximately 1 m in height were selected from the forest of the Dinghu Mountain National Natural Reserve (112°30′39″–112°33′41″E; 23°09′21″–23°11′30″N). The plants with eighteen biological replicates were grown with garden soil (pot diameter: 28 cm) in the biological garden of South China Normal University, Guangzhou, China. To simulate the phenomenon of *M. micrantha* losing its support after climbing to the top of the tree, an iron wire (diameter: 0.8 mm) was used as a support. After climbing up to a height of 70 cm along the wire perpendicular to the ground, the *M. micrantha* seedlings of the turning group were manually pulled to the iron wire horizontal to the ground to continue growing (that is, the upper part of the main stem changed from vertical to horizontal, known as turning growth). This process only changed the growth direction of the main stem and did not cause mechanical damage to the tissue. Using *M. micrantha* that always grows vertically along the wire as the control group and manual traction turning as the starting treatment, for a period of 20 days, with 10 biological replicates in each group. Different parts of *M. micrantha* main stems (CK: top site of main stem under erect growth treatment, UP: upper part of main stem under turning growth treatment, TP: turning part of main stem under turning growth treatment) were collected as materials for physiological indicators and transcriptome sequencing.

### Determination of the net photosynthetic rate

A LI-6800 Portable Photosynthesis System (LI-COR, Inc., USA) was used to measure the net photosynthetic rate (*P*_n_) of fully expanded mature leaves of *M. micrantha* in the morning (9:00–11:00) on sunny days with PPFD at 800 µmol m^− 2^ s^− 1^. The ratio of red and blue light of the irradiance in the leaf measurement chamber was set at 9:1; the mean relative air humidity was 65%, and the corresponding mean temperature was 30 °C.

### Determination of plant hormone, soluble sugar and T6P contents

Fresh stems of *M. micrantha* (0.1 g) were weighed and ground with 0.5 mL phosphate buffered saline (PBS) (50 mM, pH = 7.4) on ice. The grinding solution was centrifuged at 4 °C for 20 min at 5,000 × *g*, and the supernatant was the hormone and T6P extract. The hormone and T6P contents were determined using a plant ELISA Kit (Zike, Shenzhen, China). The extraction and determination of sucrose, glucose and fructose were performed according to the relevant assay kit (Keming, Suzhou, China).

### Total RNA extraction

The total RNA of the samples of *M. micrantha* under different treatments was extracted with a general plant total RNA extraction kit. The extraction steps were performed according to the kit instructions. Agarose gel electrophoresis was used to detect the integrity of the RNA. The quality of the sample RNA (OD260/280 ratio) was detected by an ultraviolet spectrophotometer, and poor-quality samples were re-extracted.

### Construction of the sequencing library and second-generation sequencing

After the RNA samples were qualified, the mRNA of eukaryotes was enriched by magnetic beads with oligo (dT). The mRNA was then broken into short segments by adding fragmentation buffer. Using mRNA as a template, a strand of cDNA was synthesized with six-base random primers. Then, we added the buffer, dNTPs and DNA polymer I to synthesize two-strand cDNA. Finally, the double-stranded cDNA was purified by AMPure XP beads. The end of the purified double-stranded cDNA was repaired, and a tail was added and sequenced. The fragment size was selected by AMPure XP beads, and the final cDNA library was obtained by PCR enrichment. The constructed library used Agilent 2100 to detect the length of the inserted fragments and the library was sequenced on an Illumina HiSeq™ platform after passing the quality test.

### Quality control of sequencing data and identification of differentially expressed genes (DEGs)

The raw data obtained from transcriptome sequencing were filtered to remove sequences with low quality, joint contamination and high content of unknown base N, resulting in high-quality data (clean data). These clean reads were mapped to the reference genome of *M. micrantha* (SRR8835135, SRR8835136 and SRR8835137 for the *M. micrantha* genomic Illumina DNA data) using TopHat v. 2.1.0 with the default settings [20]. The software Htseq v.0.9.1 was used to quantify the expression levels of transcripts and genes, and the corresponding TPM value of each gene was obtained [21]. In addition, DEseq2 software was used to analyse the differences in gene expression among the samples [22]. |log2Fold Change| >1 and *P* value < 0.05 were used as screening conditions for differentially expressed genes (DEGs) between the treatment group and the control group.

### KEGG enrichment analysis of DEGs

In organisms, different genes coordinate with each other to perform their biological functions. The most important biochemical metabolic pathways and signal transduction pathways involved in the differential expression of genes can be determined by significant pathway enrichment. The Kyoto Encyclopedia of Genes and Genomes (KEGG) is a database for the systematic analysis of gene functions and genomic information. It helps researchers study the whole network of genes and gene expression information. We used KOBAS (2.0) software to perform the pathway enrichment analysis [23].

### Detection of gene expression by quantitative real-time PCR (qRT‒PCR)

Total RNA of *M. micrantha* stems was extracted using a Plant Total RNA isolation kit (Huayueyang) and synthesized to complementary DNA with the M-MLV reverse transcriptase kit (Takara). qRT‒PCR was performed with a Bio-Rad CFX96 real-time PCR system (CFX96, Bio-Rad, USA) and Taq Pro Universal SYBR qPCR Master Mix Kit (Vazyme). The relative expression of genes was analysed by the 2^−∆∆CT^ method [24]. The forward and reverse primers corresponding to the internal reference gene and the target gene are shown in Table S1.

### Statistical analysis

Statistical significance was determined by one-way ANOVA followed by Duncan’s post hoc test or Student’s t test using SPSS Statistics 19.0 (IBM, NY, USA). Means were considered to be significantly different at the level *P* < 0.05. Sigmaplot 12.5 (Systat Software Inc., USA) was used to conduct linear regression analysis and plot the data. All data are shown as the means ± standard errors (S.E.).

## Result

### Growth rate and phenotype of *M. Micrantha* and trees during the climbing process

**Fig. 1:**
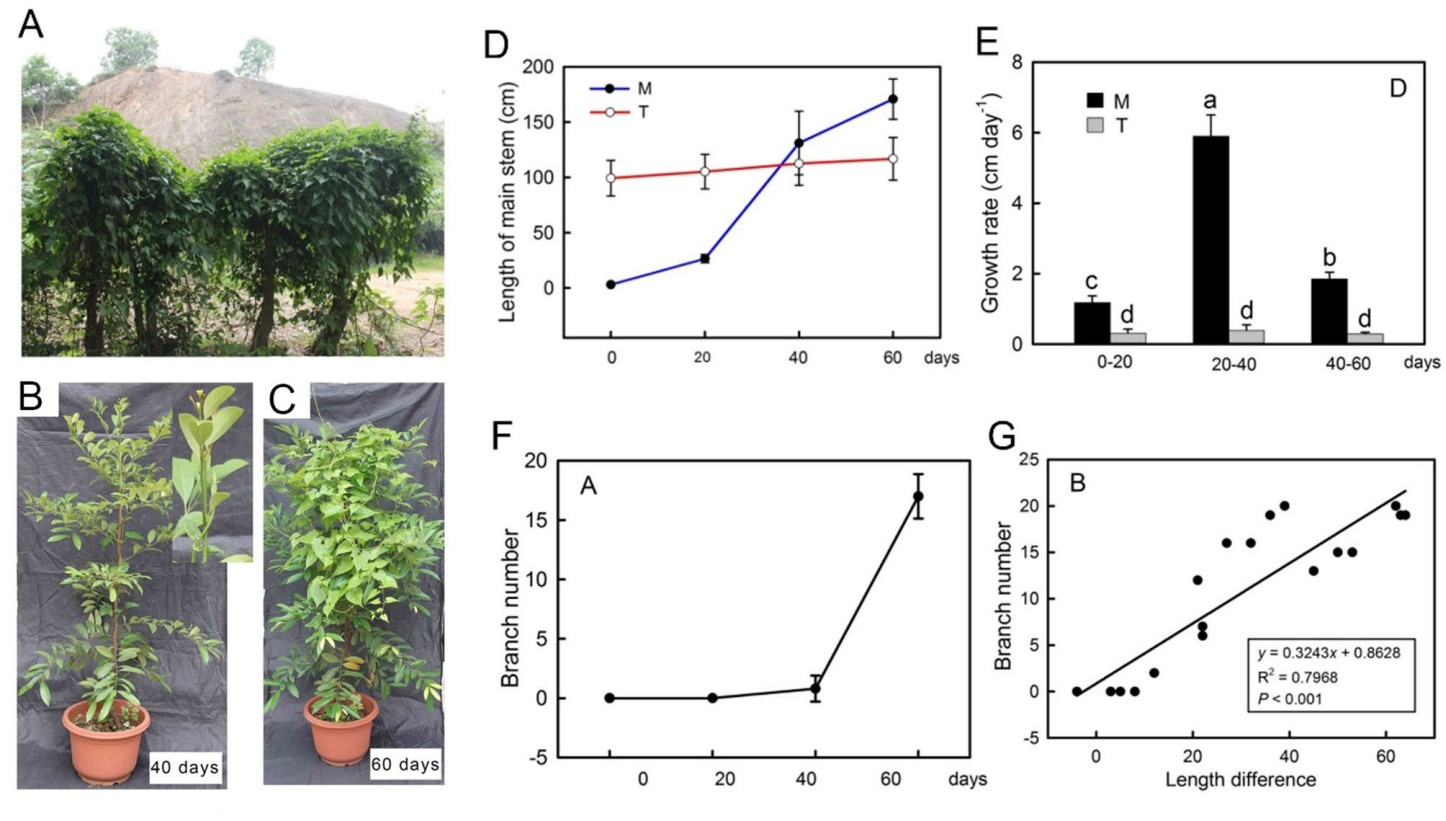
Growth phenotype of *M.micrantha* and trees (*C. concinna*). (**A**) Phenotype of *M. micrantha* covering trees in its natural state. (**B**, **C**) 40 and 60 days of *M. micrantha* and trees growing together. (**D**) Changes in the main stem length (*n* = 18). (**E**) Growth rates of *M. micrantha* and trees main stems (*n* = 18). (**F**) The change in the number of branches after the transplantation of *M. micrantha* (*n* = 18). (**G**) The relationship between branch number and the difference in length between *M. micrantha* and the tree main stems (*n* = 18). The error bars represent standard errors for eighteen biological replicates. Different letters (a, b, c…) on the bar designate statistically reliable differences of the means at *p* < 0.05

*M. micrantha* can completely cover the tree canopy by its natural growth in the field (Fig. 1A). The seedlings of *M. micrantha* were planted and grown together with the native dominant tree species *C. concinna* to simulate the climbing process of *M. micrantha* under the forest. The results showed that the growth rate of the *M. micrantha* main stem was significantly faster than that of the trees (Fig. 1E). When *M. micrantha* and trees grew together for 0 days, *M. micrantha* was only approximately 3 cm tall, while the height of the trees was approximately 100 cm, and trees were significantly taller than *M. micrantha*. After 40 days of growth, there was no significant difference in height between *M. micrantha* and the trees (Fig. 1B, D). Afterwards, the main stem length of *M. micrantha* was higher than that of the trees over time, the upper part of the stem lost the support to climb up and then turned and crawled, and the number of branches increased significantly with the increase in days. After 60 days of growth, the main stem length of *M. micrantha* was significantly longer than that of the trees, and a large number of *M. micrantha* branches and leaves had been formed near the tree canopy (Fig. 1C, D, F). There was a significant positive correlation between the number of branches and the length difference in the main stem between *M. micrantha* and the trees (*P* < 0.001) (Fig. 1G).

### Phenotype of *M. Micrantha* and changes in plant hormones, soluble sugars and T6P content under turning treatment

**Fig. 2:**
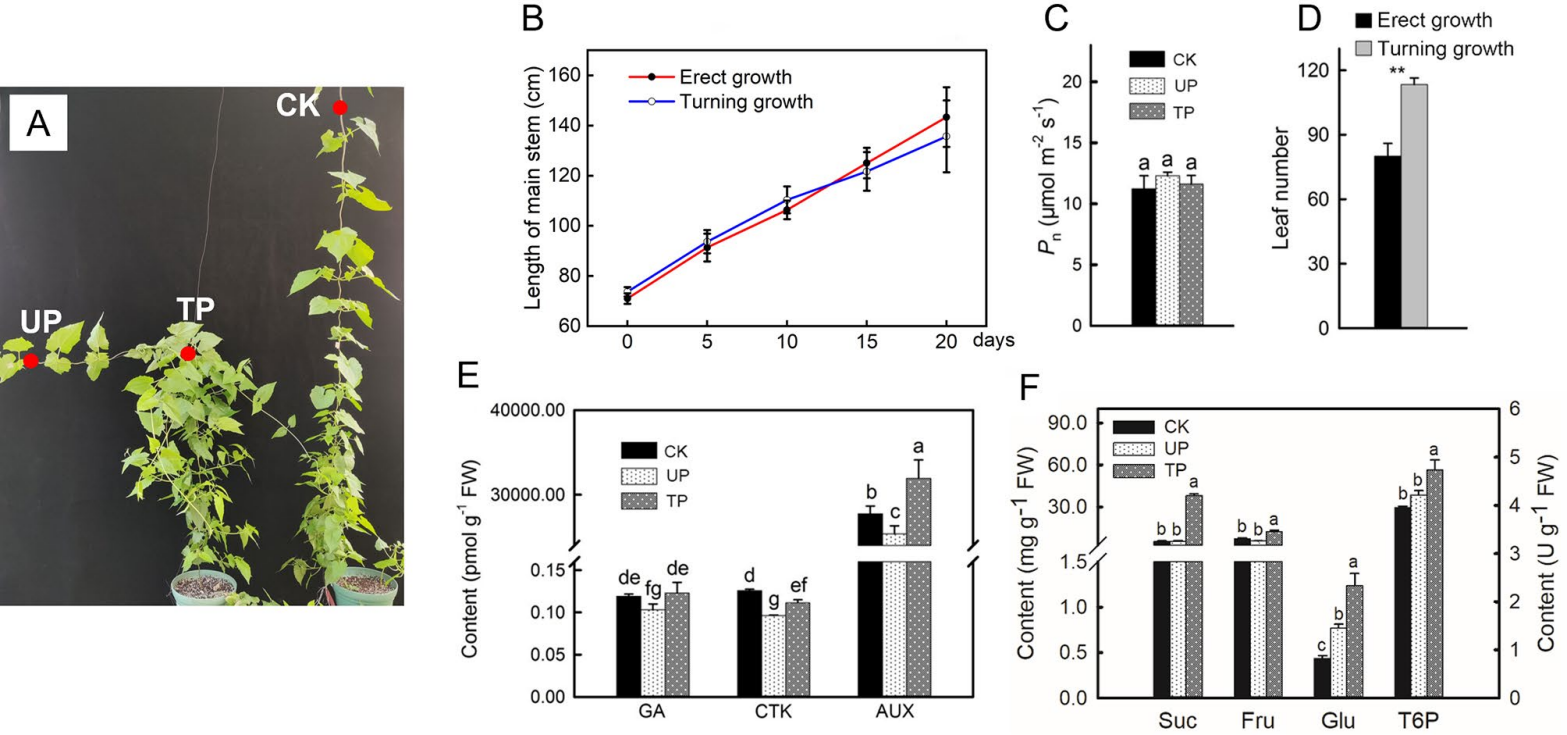
Main stem growth rate, leaf number, hormone content, sugar content and T6P content of *M. micrantha*. (**A**) The phenotype of *M. micrantha* under erect (right) or turning (left) growth treatment. (**B**) Main stem length of *M. micrantha* under different treatments (*n* = 10). (**C**) The net photosynthetic capacity of leaves in different parts of *M. micrantha* (*n* = 10). (**D**) Leaf number of *M. micrantha* under different treatments (*n* = 10). (**E**)The hormones content in different parts of *M. micrantha* stems (*n* = 6), GA: gibberellin, CTK: cytokinin, IAA: auxin. (**F**) Sugars and T6P content of different parts of *M. micrantha* stems (*n* = 3), the left vertical axis represents sucrose (Suc), fructose (Fru) and glucose (Glu) content, and the right vertical axis represents trehalose-6-phosphate (T6P) content. CK: top site of *M. micrantha* stem under erect growth treatment, UP: upper part of *M. micrantha* stem under turning growth treatment, TP: turning part of *M. micrantha* stem under turning growth treatment. The error bars represent standard errors for three to ten biological replicates. Different letters (a, b, c…) on the bar designate statistically reliable differences of the means at *p* < 0.05. Asterisks indicate different significant differences (** *P* < 0.01) according to two-sided Student’s *t*-tests

To simulate the branching process of *M. micrantha* after climbing to the top of the trees, *M. micrantha* was treated with turning growth. As shown in the phenotype of Fig. 2A, compared to erect growth, *M. micrantha* produced more branches near the turning part of the main stem after turning treatment. The relevant indicators of the top site of the main stem under erect growth (CK), the upper part of the main stem under turning growth (UP), and the turning part of the main stem under turning growth (TP) were further measured. The results showed that the growth rate of the main stem of *M. micrantha* was not significantly affected by the different treatments (Fig. 2B). The *P*_n_ of leaves at different positions was not significantly different (Fig. 2C), but the leaf number of *M. micrantha* with turning growth was significantly higher than that of *M. micrantha* with erect growth (Fig. 2D). The hormone content in the stem of *M. micrantha* was measured. The results showed that there was no significant difference in gibberellin content between TP and CK. The cytokinin content was significantly lower at TP than at CK. The auxin content was significantly higher in TP than in CK. In the turning group, the three hormone contents at TP were significantly higher than those at UP (Fig. 2E). The contents of three main soluble sugars (sucrose, glucose, fructose) and trehalose-6-phosphate (T6P) in the stem were determined, and the results showed that the contents of sucrose, fructose, glucose and T6P at TP were significantly higher than those at CK and UP (Fig. 2F).

### KEGG enrichment analyses of DEGs in *M. micrantha* under turning treatment

**Fig. 3:**
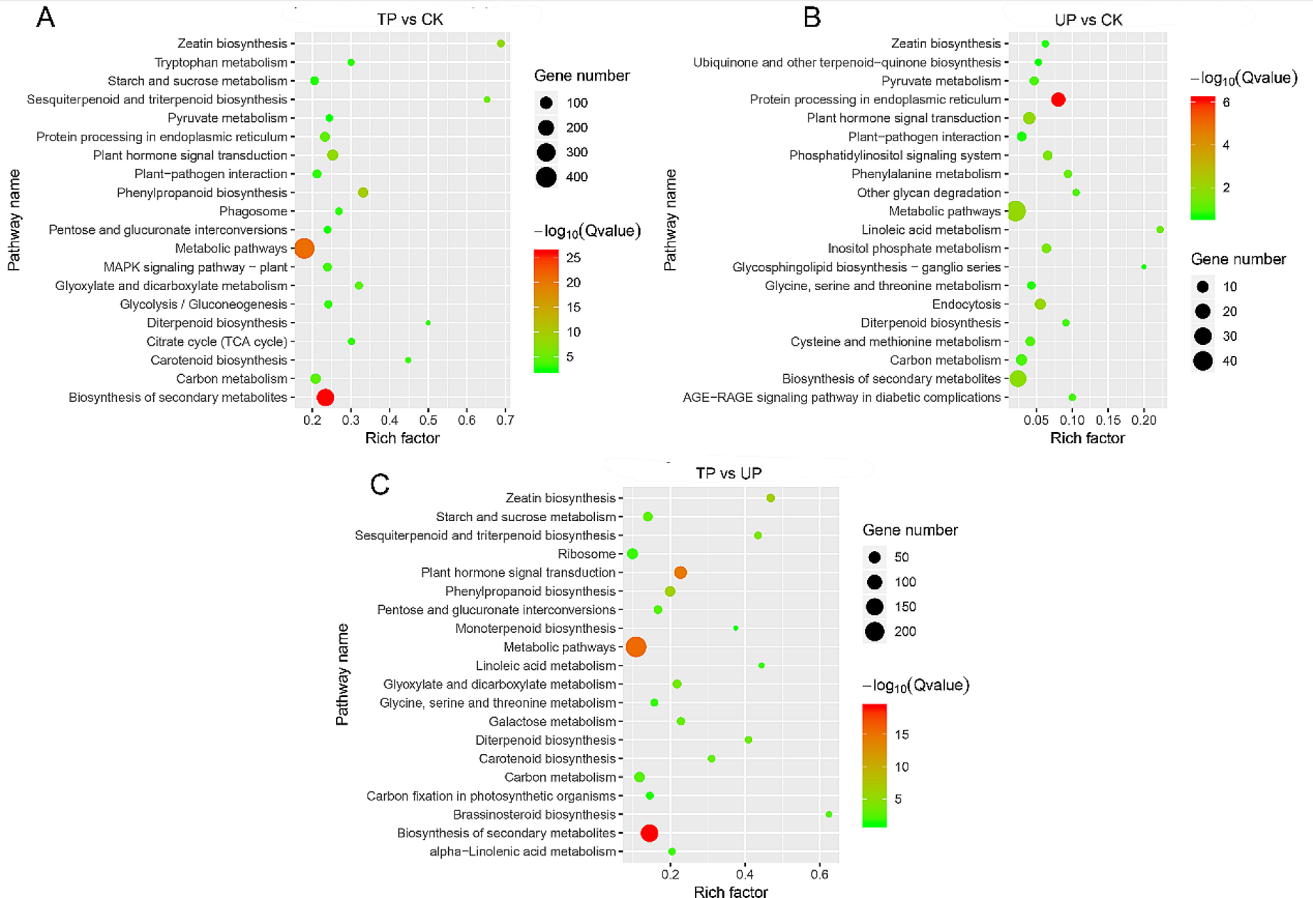
KEGG enrichment analysis in *M. micrantha* stem under different treatment. (**A**) Bubble map of DEGs in TP group vs. CK group. (**B**) Bubble map of DEGs in UP group vs. CK group. (**C**) Bubble map of DEG in TP group vs. UP group. CK: top site of *M. micrantha* stem under erect growth treatment, UP: upper part of *M. micrantha* stem under turning growth treatment, TP: turning part of *M. micrantha* stem under turning growth treatment. Permission has been obtained from Kanehisa laboratories for using KEGG database [25–27]

A total of three parts (CK, UP, TP) of the main stem in *M. micrantha* under the two treatments were analysed by reference transcriptome sequencing. The KEGG enrichment degree was determined by the P value, and the pathways with significant enrichment (*P* value < 0.05) were selected for analysis. The 20 pathways with the highest enrichment degree are shown in Fig. 3. The results showed that compared with CK and UP, DEGs in TP were significantly enriched in the starch and sucrose metabolism pathway (*P* value = 4.70E-04 and *P* value = 3.84E-04) and the plant hormone signal transduction pathway (*P* value = 9.82E-10 and *P* value = 9.24E-17) (Fig. 3A, C). Compared with CK, DEGs in UP were significantly enriched in the plant hormone signal transduction pathway (*P* value = 1.21E-03) (Fig. 3B). The results indicated that the turning treatment had significant effects on the ‘starch and sucrose metabolism’ and ‘plant hormone signal transduction’ pathways in different parts of the *M. micrantha* main stem.

### Expression levels of genes related to starch and sucrose metabolism pathways and SWEET transporters in *M. micrantha* under turning treatment

**Fig. 4:**
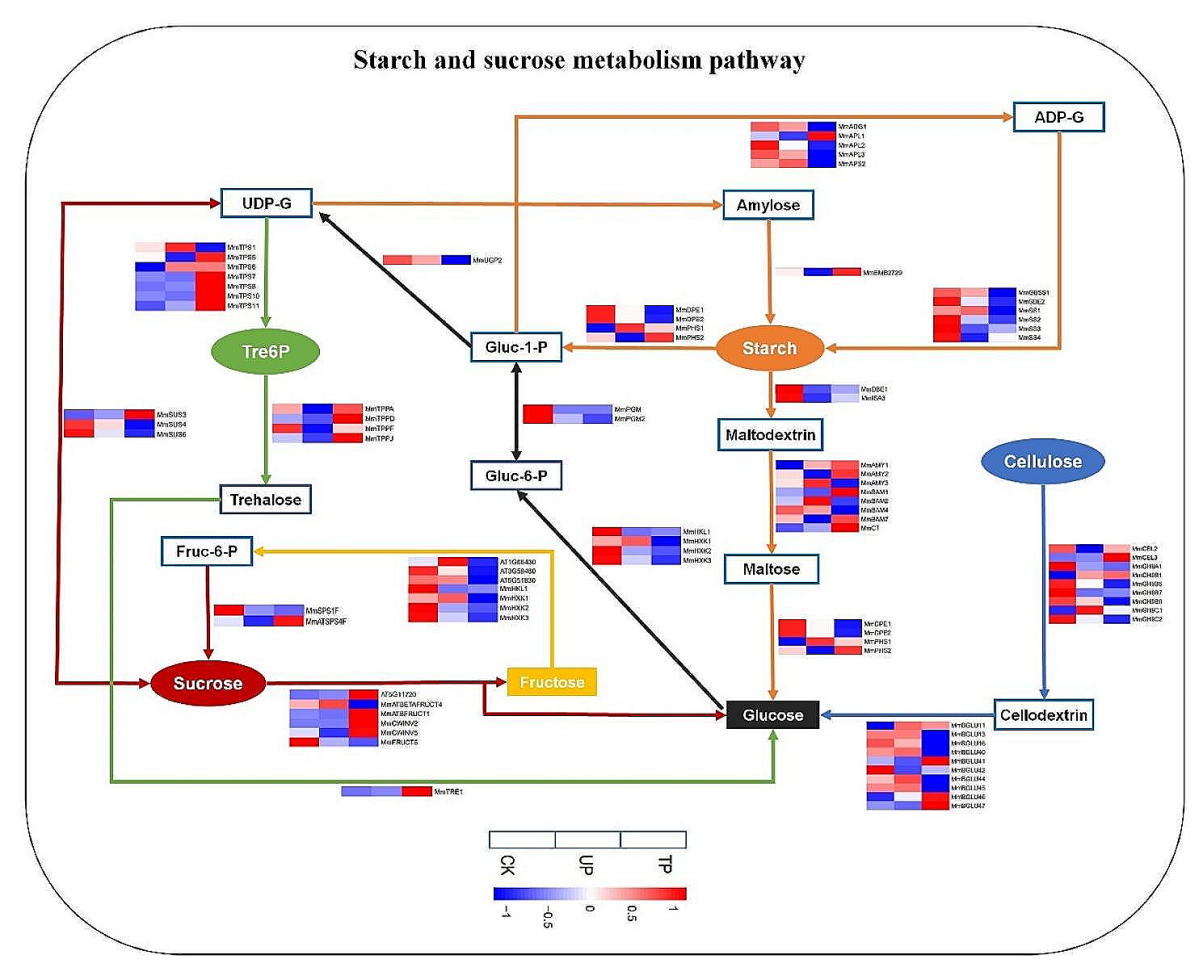
Heat map of annotated genes in starch and sucrose metabolism pathway. The starch synthesis and hydrolysis process, sucrose synthesis and hydrolysis process, cellulose hydrolysis process, trehalose metabolism, fructose and glucose hydrolysis process were marked in orange, red, blue, green, yellow and black arrows, respectively. The TPM value was used to create heatmaps. CK: top site of *M. micrantha* stem under erect growth treatment, UP: upper part of *M. micrantha* stem under turning growth treatment, TP: turning part of *M. micrantha* stem under turning growth treatment

Gene expression profiles associated with starch and sucrose metabolism pathways are shown in Fig. 4. For the starch synthesis and hydrolysis process, we found that compared with CK, *MmADG1*, *MmAPL2/3*, *MmAPS2*, *MmGBSS1*, *MmSBE2*, and *MmSS1/2/3/4* (associated with starch synthesis) were downregulated at TP, and the expression levels of *MmAPL3*, *MmGBSS1* and *MmSS2* were 0.3, 0.2 and 0.5 times those at CK, respectively, while genes including *MmAMY1/2*, *MmBAM1/7*, *MmCT*, and *MmPHS1/2* (associated with starch hydrolysis) were upregulated, and the expression of *MmAMY1*, *MmCT*, and *MmPHS1* at TP was 3.6, 2.2, and 2.0 times higher than that at CK, respectively. For the genes involved in the cellulose hydrolysis process, compared to CK, *MmCEL3*, *MmGH9B1/C1* and *MmBGLU11/41/46/47* were highly expressed at TP, and the expression levels of *MmCEL3* and *MmBGLU47* at TP were 3.9 and 3.1 times those at CK, respectively. For the sucrose hydrolysis process, compared with CK, genes including *AT5G11720*, *MmATBFRUCT1* and *MmCWINV2/5* were upregulated at TP, and the expression of *AT5G11720*, *MmATBFRUCT1* and *MmCWINV2* was 23.9, 13.6, and 853.3 times that at CK, respectively. The genes involved in fructose and glucose hydrolysis, *AT1G66430*, *AT3G59480*, *AT5G51830*, *MmHKL1*, *MmHXK1/2/3*, *MmPGM/MmPGM2* and *MmUGP2*, were expressed at lower levels at TP than at CK. The expression of *AT3G59480*, *MmHXK1/2/3*, *MmPGM2* and *MmUGP2* was 0.3, 0.4, 0.2, 0.4, 0.4, and 0.5 times that of CK, respectively. For the trehalose metabolism process, we found that compared with CK, most T6P synthase (TPS) genes and T6P phosphatase (TPP) genes were upregulated at TP, and the expression of *MmTPPD/J* and *MmTPS7/10/11* was 3.4, 2.6, 2.7, 2.9, and 15.1 times higher, respectively, than that at CK (Fig. 4). Sugars will eventually be exported transporter (SWEET) proteins, which are mainly involved in the phloem unloading of plant hexose and sucrose in sink organs [28]. According to the Swiss Prot database annotation, a total of 23 SWEET proteins were found in *M. micrantha*. Further analysis of the expression level of SWEETs in *M. micrantha* stems under different treatments showed that compared to CK, the expression of 6 SWEET genes (*MmSWEET1/3/7/10_1/14_7/15_3*) at TP was upregulated, among which the expression levels of *MmSWEET7/10_1/15_3* were 2.0, 13.9, and 2.0 times higher than those at CK, respectively (Table 1).

**Table 1:** Expression levels of SWEET genes

Gene name	TPM value		
	CK	UP	TP
*MmSWEET1*	0	0.184	0.206
*MmSWEET2*	57.582	68.183	33.050
*MmSWEET3*	1.235	1.246	1.264
*MmSWEET5*	0	0	0
*MmSWEET6b_1*	0	0	0
*MmSWEET6b_2*	0	0	0
*MmSWEET7*	0.245	0	0.492
*MmSWEET9*	0	0	0
*MmSWEET10_1*	0.646	2.185	8.992
*MmSWEET10_2*	0.745	0.474	0.719
*MmSWEET14_1*	0	0	0
*MmSWEET14_2*	0	0.170	0.028
*MmSWEET14_3*	0.251	0.856	0.056
*MmSWEET14_4*	0.592	0.213	0.111
*MmSWEET14_5*	0.298	0.506	0
*MmSWEET14_6*	0	0	0
*MmSWEET14_7*	0.103	0	0.153
*MmSWEET14_8*	0	0	0
*MmSWEET15_1*	0	0	0
*MmSWEET15_2*	0	0	0
*MmSWEET15_3*	8.644	8.130	17.116
*MmSWEET16*	0.262	0.229	0.102
*MmSWEET17*	18.811	9.169	15.122

### Expression levels of genes related to plant hormone biosynthesis and signal transduction in *M. micrantha* under turning treatment

**Fig. 5:**
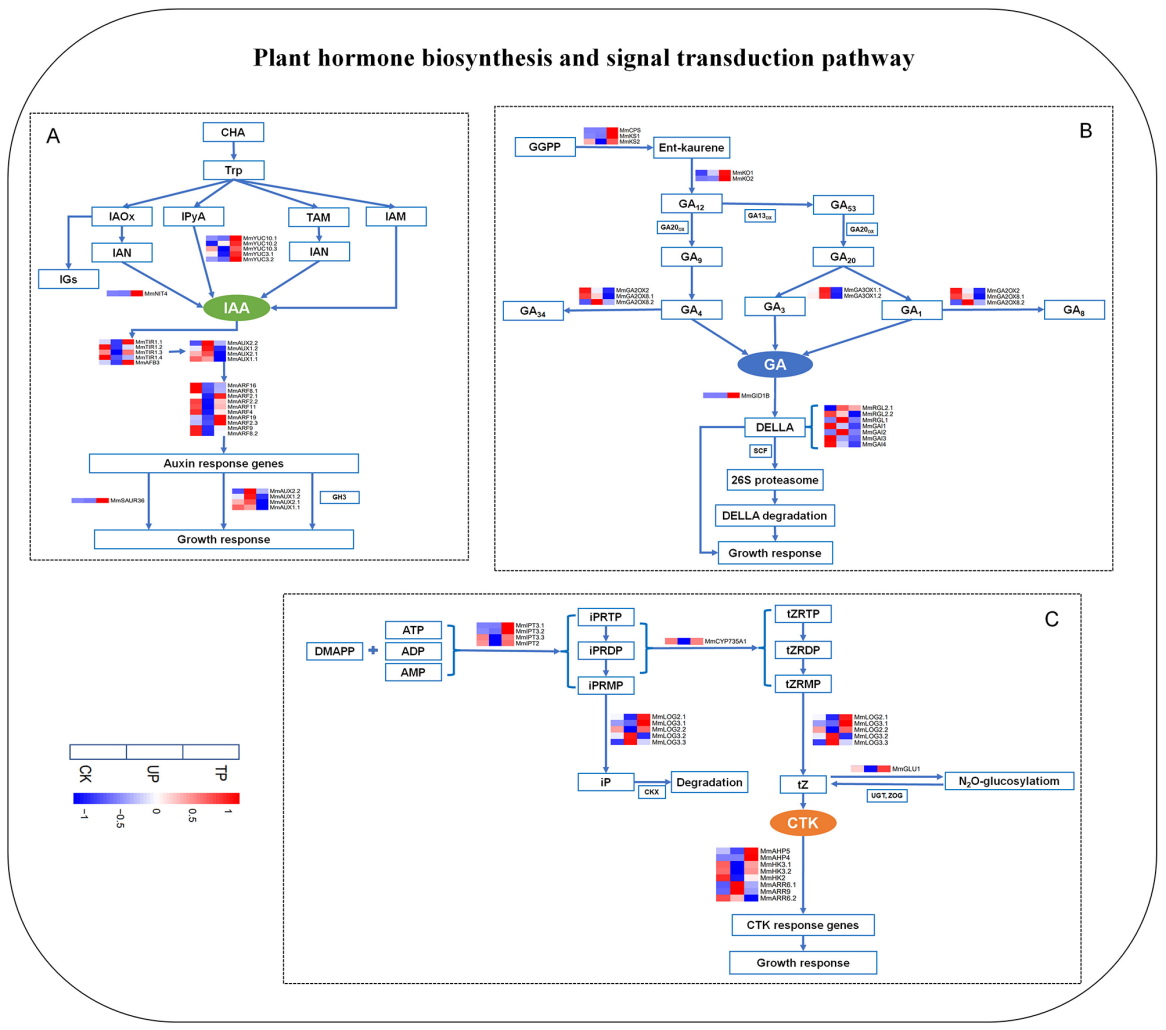
Heat map of annotated genes in plant hormone biosynthesis and signal transduction pathway. (**A**) Expression of annotated genes related to auxin (IAA) biosynthesis and signal transduction pathway. (**B**) Expression of annotated genes related to gibberellin (GA) biosynthesis and signal transduction pathway. (**C**) Expression of annotated genes related to cytokinin (CTK) biosynthesis and signal transduction pathway. The TPM value was used to create heatmaps. CK: top site of *M. micrantha* stem under erect growth treatment, UP: upper part of *M. micrantha* stem under turning growth treatment, TP: turning part of *M. micrantha* stem under turning growth treatment

The expression levels of genes associated with auxin, gibberellin and cytokinin biosynthesis and signal transduction are shown in Fig. 5. YUCCA (YUC) flavin monooxygenases and nitrilase (NIT) play positive roles in the auxin synthesis pathway and directly affect auxin synthesis. The expression of *MmYUC* and *MmNIT* was upregulated at TP compared with that at CK and UP, among which the expression of *MmYUC3.2* was 4.5 times that at CK and the expression of *MmYUC3.2/10.3* was 8.9 and 2.4 times higher than that at UP. In the auxin signalling pathway, the transport inhibitor response (TIR) and auxin response element (Aux) play negative roles, and auxin response factor (ARF) and small auxin-up RNA (SAUR) play positive roles. Compared with CK, the expression of *MmTIR1.2/1.4* and *MmAUX1.1/1.2/2.1* was downregulated at TP, while the expression of *MmARF2.1/2.3/19* and *MmSAUR36* was upregulated and the expression level of *MmSAUR36* was 5.4 times that at CK (Fig. 5A). Ent-copalyl diphosphate synthase (CPS), ent-kaurene synthase (KS), gibberellin 2-oxidase (GA2_OX_) and gibberellin 3-oxidase (GA3ox) play positive roles in the gibberellin synthesis pathway. The expression of *MmCPS*, *MmKS* and *MmKO* was upregulated at TP compared with that at UP, among which the expression of *MmCPS*, *MmKS1* and *MmKO2* was 206.4, 11.3, and 2.3 times higher than that at UP, respectively. In the gibberellin signalling pathway, RGA-LIKE (RGL) and GA-INSENSITIVE (GAI), which are involved in DELLA protein synthesis, play negative roles, while gibberellin-insensitive dwarf (GID) plays a positive role. The expression of *MmGID1B* was upregulated at TP compared with that at UP, and its expression level was 2.4 times that at UP, while *MmRGL2.1/2.2* was downregulated at TP (Fig. 5B). Isopentenyltransferase (IPT), cytochrome P450 monooxygenase 735 A (CYP735A), β-glucosidase (GLU) and LONELY GUY (LOG) play positive roles in the cytokinin synthesis pathway. The expression of *MmIPT*, *MmCYP735A*, *MmLOG2.1/2.2/3.1* and *MmGLU1* was upregulated at TP compared with that at UP, and the expression of *MmIPT3.1/3.2/3.3*, *MmCYP735A*, *MmLOG2.1* and *MmGLU1* was 75.3, 2.1, 3.6, 2.3, 2.3, and 2.0 times that at UP, respectively. In the cytokinin signalling pathway, type-A response regulator (ARR) plays a negative role, and Arabidopsis histidine kinase (AHK) and histidine phosphotransfer proteins (AHP) play positive roles. Compared with UP, the expression of *MmAHP* and *MmHK* was upregulated, while *MmARR* was downregulated at TP, among which the expression of *MmAHP4/5*, *MmHK3.1* and *MmARR6.2/9* was 292.6, 2.5, 2.5, 0.4, and 0.5 times that at UP, respectively (Fig. 5C).

### Real-time qPCR assay of sugar metabolism, plant hormone synthesis and signalling pathways

**Fig. 6:**
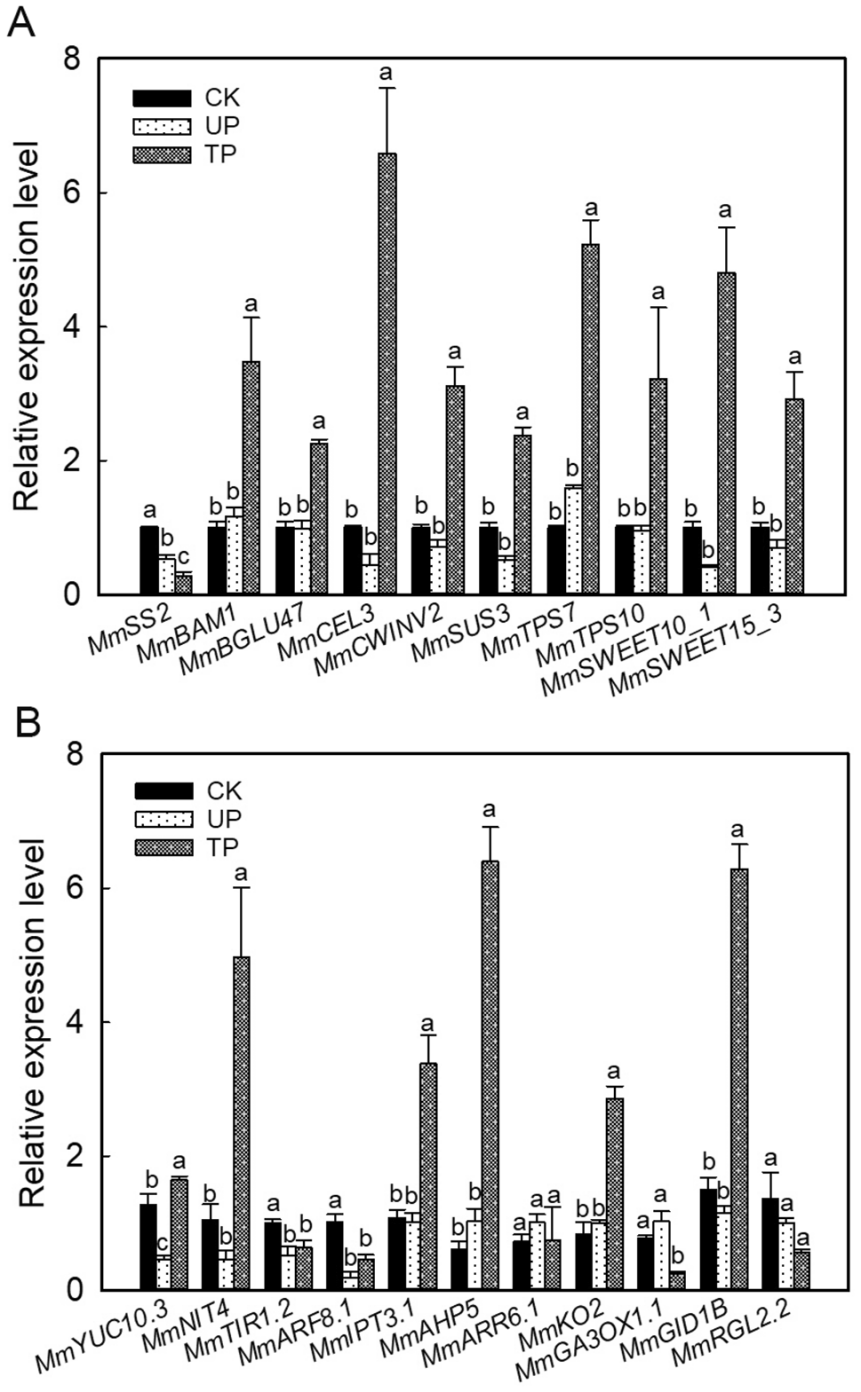
Relative expression of associated genes in (**A**) starch and sucrose metabolism and SWEET proteins and (**B**) auxin, cytokinin and gibberllin biosynthesis and signal transduction pathways. The error bars represent standard errors for three biological replicates. Different letters (a, b, c…) on the bar designate statistically reliable differences of the means at *p* < 0.05. CK: top site of *M. micrantha* stem under erect growth treatment, UP: upper part of *M. micrantha* stem under turning growth treatment, TP: turning part of *M. micrantha* stem under turning growth treatment

To further verify the expression profiles of genes related to sugar metabolism and plant hormone pathways in RNAseq, 8, 2 and 11 genes related to sucrose and starch metabolism pathways, SWEET proteins, and three plant hormone synthesis and signalling pathways were selected for qPCR assays to determine their relative expression levels. The results showed that compared to CK, the relative expression levels of the *MmSS2*, *MmBAM1*, *MmBGLU47*, and *MmCEL3* genes involved in starch and cellulose metabolism at TP were 0.3, 3.5, 2.2, and 6.6 times those at CK, respectively. The relative expression of the genes *MmCWINV2*, *MmSUS3*, *MmTPS7*, and *MmTPS10* involved in sucrose metabolism and T6P synthesis at TP was 3.1, 2.4, 5.2, and 3.2 times higher than that at CK, respectively. The relative expression of the sugar transport carrier genes *MmSWEET10_ 1* and *MmSWEET15_ 3* at TP was 4.8 and 2.9 times higher than that at CK, respectively (Fig. 6A). In the auxin synthesis and signal transduction pathway, the expression levels of *MmYUC10.3*, *MmNIT4*, *MmTIR1.2*, and *MmARF8.1* at TP were 1.7, 5.0, 0.6, and 0.5 times those at CK, respectively. Compared with UP, the relative expression levels of *MmIPT3.1*, *MmAHP5* and *MmARR6.1* in the cytokinin synthesis and signalling pathways at TP were 3.4, 6.4 and 0.8 times those at UP, respectively. The relative expression levels of *MmKO2*, *MmGA3OX1.1*, *MmGID1B*, and *MmRGL2.2* in the gibberellin synthesis and signalling pathways at the TP were 2.9, 0.2, 6.3, and 0.6 times those at the UP, respectively (Fig. 6B). The qPCR results were basically consistent with the corresponding gene expression patterns obtained by RNA-Seq.

## Discussion

The combination of rapid growth, unique resource acquisition, and climbing and dispersal adaptations gives vines a competitive advantage over self-supporting plants [29] and enables them to compete for light resources in high ecological niches by climbing tall trees. *M. micrantha*, as an invasive vine, has more exuberant stem growth habits and vegetative reproduction ability, which are key to successfully covering the canopy and invading the forest. In the experiment simulating climbing under the forest, the growth rate of the *M. micrantha* main stem was significantly higher than that of the tree as a support (Fig. 1E) and climbed to the top of the tree at 40 days (Fig. 1B). After losing upwards climbing support and growing in a turning way (40–60 days), the elongation rate of the *M. micrantha* main stem slowed, while the number of branches increased rapidly and were concentrated in the canopy (Fig. 1C, D, E, F), thus achieving the purpose of covering trees. To better understand how *M. micrantha* produced a large number of branches after turning growth, we treated the *M. micrantha* main stem as turning growth and found that turning treatment had a significant effect on the distribution of soluble sugars, T6P and plant hormones in different parts of the main stem (Fig. 2E, F) and formed a phenotype in which the branches were concentrated at the turning part of the main stem (TP) (Fig. 2A). Further combined with KEGG enrichment analysis, it was found that DEGs in TP vs. CK and TP vs. UP were both enriched in starch and sucrose metabolism pathways and plant hormone signal transduction pathways (Fig. 3A, C); thus, the branching process at TP is closely related to the regulation of sugars and plant hormones.

### The accumulation of sugars and T6P at the TP promotes the release of nearby dormant buds

**Fig. 7:**
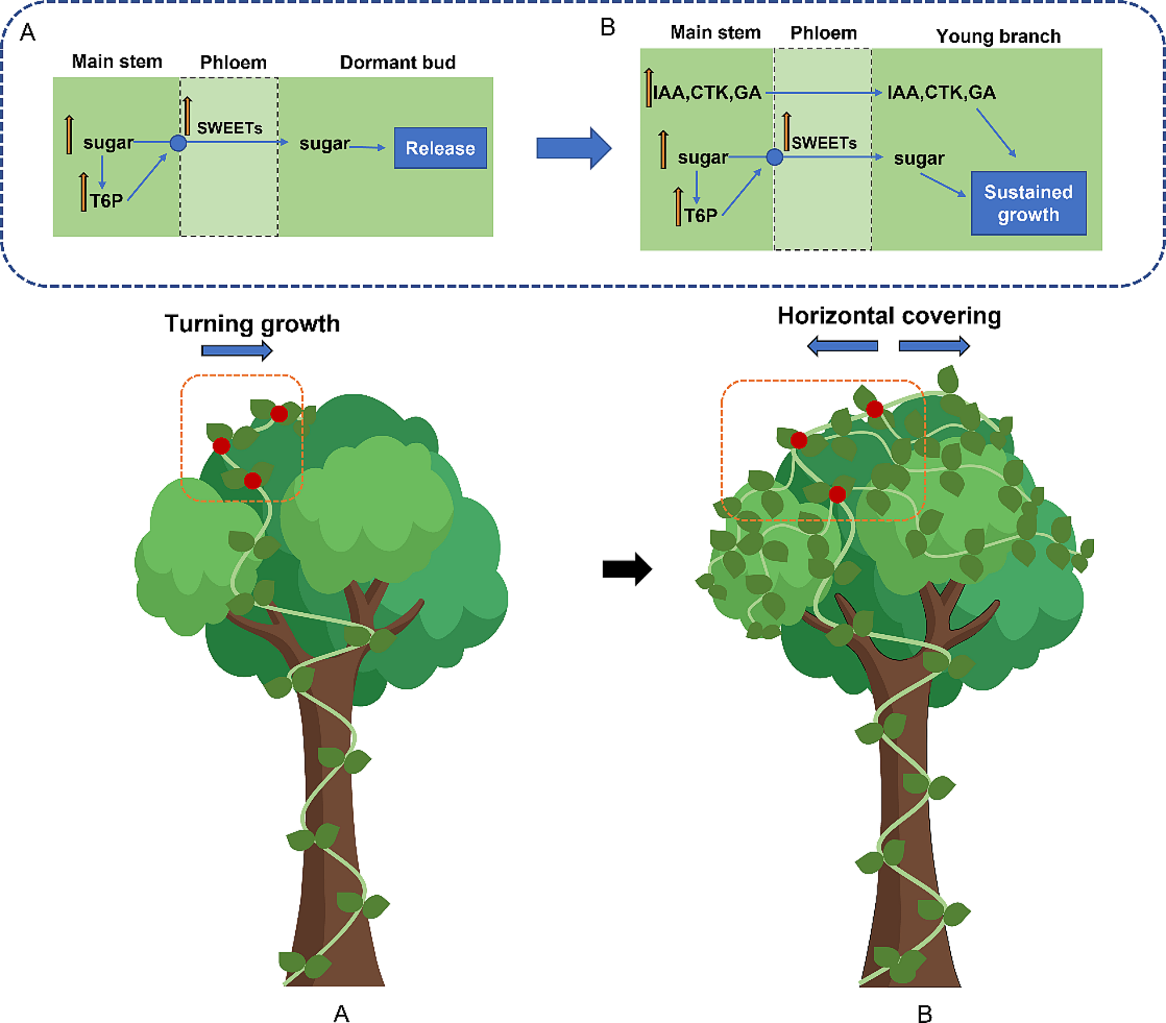
Hypothetical invasion strategies of *M. micrantha* covering trees. (**A**) Hypothetical regulatory process of dormant buds release after turning growth of *M. micrantha*. (**B**) Hypothetical regulatory process of sustained growth from buds to branches during covering trees of *M. micrantha*

The number of branches of perennials usually depends on whether dormant buds are released and whether they enter sustained growth, while the key release process is inhibited by apical dominance [9, 30]. From a trophic point of view, buds are regarded as sink organs that need to import sugars to meet their metabolic demand and support their growth, and the growth ability of buds is also reflected in their sink strength, that is, the ability to acquire and use sugars [31–33]. Therefore, recent studies have gradually proposed that an increase in sugar supply is a sufficient and necessary condition for lateral bud early release from apical dominance [6, 7]. Meanwhile, the sucrose-specific signalling pathway mediated by trehalose-6-phosphate (T6P) was also confirmed to be involved in the early stage of dormant bud release from pea [34]. The results of the turning experiment showed that the contents of three main soluble sugars (sucrose, fructose, glucose) and T6P at TP were significantly increased compared with those at CK and UP (Fig. 2F). Transcriptome data were used to analyse the gene expression of starch and sucrose metabolism pathways at TP under turning treatment. Compared to CK, the expression levels of genes involved in starch hydrolysis (*MmAMY1*, *MmCT* and *MmPHS1*), cellulose hydrolysis (*MmCEL3* and *MmBGLU47*) and sucrose hydrolysis (*AT5G11720*, *MmATBFRUCT1* and *MmCWINV2*) were obviously upregulated at TP, while those involved in glucose and fructose hydrolysis processes, such as *AT3G59480*, *MmHXK1*/*2*/*3*, *MmPGM2* and *MmUGP2*, were downregulated, which indicated that the accumulation of the main decomposition products of sugars, glucose and fructose, was promoted by intensifying the decomposition of polysaccharides (starch and cellulose) and disaccharides (sucrose) and slowing the decomposition of monosaccharides (glucose and fructose) at TP (Fig. 4). Sucrose, as one of the photosynthetic products, is mainly synthesized in the leaves and then transported to other plant organs through the stem phloem. The turning treatment may affect the downwards transportation of sucrose in the main stem, leading to a high level of sucrose accumulation at the TP. In addition, a study showed that supplying sucrose to stem node explants in vitro triggered a concentration-dependent increase in the T6P content of the buds, which was highly related to the initial release and growth rate of lateral buds [34]. T6P is synthesized by T6P synthase (TPS) and dephosphorylated to trehalose by T6P phosphatase (TPP). The expression heatmap of genes associated with trehalose metabolism showed that the expression of TPS genes at TP was significantly higher than that of CK, so the accumulation of sucrose at TP might increase the level of T6P here by promoting the expression of TPS genes (Fig. 4). As such, the obvious accumulation of sucrose, fructose, glucose and T6P at TP may provide a high level of sugar supply and signal regulation for the early release and outgrowth of dormant buds near TP. At this point, TP serves as a source to input sugar into dormant buds, which requires the unloading process mediated by sugar transporters in the phloem of the sink organ, such as the sugar transport carrier SWEET, which is mainly responsible for the transportation of hexose and sucrose [28, 35]. Research has suggested that a high level of T6P may regulate the release and growth of dormant buds by upregulating the expression of SWEETs to enhance the supply of sugar to buds [10]. In this study, we found that compared to CK, the expression of 6 SWEET genes (*SWEET1*/*3*/*7*/*10_1*/*14_7*/*15_3*) was upregulated at TP, indicating the active process of transporting sugar from TP to nearby dormant buds (Table 1). Consequently, based on the above physiological and transcriptome results, we hypothesized the regulation strategy of *M. micrantha* in the early release stage of dormant buds after reaching the tree canopy: when it climbed to the top of the tree, the lack of support caused its main stem to be turning growth, affecting the transport of sucrose, and the process of sugar metabolism at TP was intensified, leading to the accumulation of soluble sugars and T6P. The strong sugar source at the TP and the active expression of sugar transporter SWEET genes enhanced the sugar supply to dormant buds, which led to the release and growth of nearby dormant buds, thereby initiating the process of forming a large number of branches at the TP of *M. micrantha* (Fig. 7A). Simultaneously, some problems are worthy of further study: why is sugar metabolism enhanced at TP? How do sugar and T6P exert energy and signalling effects on the release of dormant buds? Which sugar is the main evoked signal of this process?

### The accumulation of plant hormones at the TP promotes the sustained growth of dormant buds and regulates them into branches together with sugars

Once the dormant buds of plants are released, they will continue to grow into branches or remain dormant [36]. A decapitation experiment in pea suggested that auxin coordinated with other plant hormones, such as cytokinins, played an important role in regulating sustained growth at the later stage of lateral buds [8, 9, 37]. Recently, a study has suggested that an increase in auxin levels can promote sustained bud growth by promoting gibberellin synthesis in buds [5]. The results of the turning experiment showed that the IAA, CTK and GA contents at CK and TP under erect and turning growth, respectively, were significantly higher than those at UP under turning growth, and the IAA level at TP was also significantly higher than that at CK (Fig. 2E). The RNA-Seq results also showed that compared with UP, the genes in TP that played active roles in three plant hormone synthesis pathways (including *MmYUC3.2*, *MmNIT4*, *MmIPT3.1*, *MmGLU1*, *MmCPS*, *MmKS1*, etc.) were all upregulated. Positive regulators (including *MmSAUR36*, *MmAHP4*, *MmGID1B*, etc.) and negative regulators (including *MmTIR1.2*, *MmARR6.2*, *MmRGL2.1*, etc.) related to three plant hormone signalling pathways at TP were upregulated and downregulated, respectively, compared with UP (Fig. 5). The results indicated that the biosynthesis and downstream transduction of IAA, CTK and GA at TP were active, which was consistent with its high content level. In comparison with CK, the positive and negative regulatory genes (such as *MmYUC10.3*, *MmARF2.1* and *MmTIR1.4*) of IAA synthesis and signal transduction at TP also showed an obviously up- or downregulated trend, which further verified the physiological results of the highest content of IAA at TP (Figs. 2E and 5A). Plant hormones are involved in the regulation of plant growth and development, among which three main hormones, including auxin, cytokinin and gibberellin, are involved in the regulation of cell division, elongation and branching configuration [38–41]. As a result, *M. micrantha* uses the different distributions of plant hormones in the main stem to implement different regulation strategies for erect or turning treatments, resulting in significant branching phenotypic differences. Under the condition of always having support (erect growth), a higher content of GA, CTK and IAA in CK is an important factor in maintaining its apical dominance and continuously climbing upwards. In contrast to the distribution strategy of erect growth, plant hormones no longer only focused on the elongation of UP but were more distributed at TP due to the weakening of apical dominance after turning treatment of *M. micrantha*. After the dormant buds near the TP were successfully released through the high level of sugar supply from the main stem, a possible regulatory mechanism was proposed based on the canalization model and the second messenger model theories of auxin regulating apical dominance. One proposed regulatory mechanism is that the accumulation of CTK at the TP may promote the sustained growth of buds by entering the bud to promote the efflux of IAA in the buds. Another possible mechanism is that the accumulation of IAA may promote the sustained growth of buds by regulating the synthesis of CTK and GA, which enter the bud to regulate the outgrowth of the bud, hence forming a large number of branches and finally successfully covering the trees (Fig. 7B). During this process, research has also proposed that sugars serve as energy and signal entities that participate in the regulation of bud growth in the later stage [9]. It would be worth investigating the reasons for the accumulation of plant hormones at TPs and the molecular mechanism by which auxin crosstalks with sugar-related pathways to regulate hormone and sugar levels in buds to promote their sustainable growth.

In summary, we put forward a hypothetical model for the invasion strategy of *M. micrantha* covering the tree canopy quickly: after rapidly climbing to the top of the tree, the loss of climbing support in the canopy led to the turning growth of the main stem. The accumulation of sugars and T6P at TP first promoted the release of dormant buds near the TP, then the accumulation of plant hormones regulated the bud to enter sustained growth, and in the later stage, together with sugar, maintained the continuous growth of the buds to become branches. A large number of branches wound and climbed horizontally in the canopy and finally successfully covered the trees and occupied the habitat. The results of this branching hypothesis model indicated that the accumulation and transportation of sugars, as well as the regulation of plant hormones, were important reasons for the formation of a large number of branching in *M. micrantha*. Therefore, future research on inhibitors of sugar synthesis or transport, as well as auxin synthesis or polar transport, can serve as a control measure to inhibit the growth and branching of *M. micrantha*. At the same time, spray-induced gene silencing (SIGS) can produce RNAi silencing effect without introducing genetic modification [42], which could be used to silence the potential candidate target genes in the transcriptome data of this study to achieve the purpose of genetic prevention and control of *M. micrantha*.

### Electronic supplementary material

Below is the link to the electronic supplementary material.

Supplementary Material 1

## Data Availability

The library was sequenced using the Illumina HiSeq™ platform. Raw Illumina sequences and assembled sequences are available in the NCBI Sequence Read Archive (accession number: CK1-CK3:SRR10521023-SRR10521025, UP1-UP3: SRR10521020-SRR10521022, TP1-TP3: SRR10521017-SRR10521019). Other data supporting the findings of this study are available within the paper and its supplementary file.
