# Supplementary material for: Why can Mikania micrantha cover trees quickly during invasion?
Source: BMC Plant Biol. 2024 Jun 7;24:511. doi: 10.1186/s12870-024-05210-5 (PMC11157800; doi:10.1186/s12870-024-05210-5)
Supplement: Supplementary file 1 — Supplementary Material 1 [file 12870_2024_5210_MOESM1_ESM.docx]

**Table S1.** Primer sequences of relevant genes

| **Gene name** | **Sequence** |
| --- | --- |
| *18s rRNA* | F: 5’-GTCGGGGGCATTCGTATTTC-3’  R: 5’-CGGCATCGTTTATGGTTGAG-3’ |
| *MmSUS3* | F: 5’- CTCACAGTTGTGGAGGCCAT-3’  R: 5’- TGTGGAAACCCGAAACACCA-3’ |
| *MmTPS7* | F: 5’- CTGAGCCGGTCATGAACCTT-3’  R: 5’- AGCCTGGTCTAAGCCGAAAC-3’ |
| *MmTPS10* | F: 5’- GGTTTGGCTGCTGAACATGG-3’  R: 5’- TGCTCGAACCATCAGTTGCT-3’ |
| *MmATBFRUCT1* | F: 5’- AAATACACCGGCAACCCGAT-3’  R: 5’- CCAGTGGCTGTTCATACCGT-5’ |
| *MmCWINV2* | F: 5’- GGGGGAATATTGTGTGGGCA-3’  R: 5’- GACTTGGTGACCAGGGTCTG-3’ |
| *MmSWEET10_1* | F: 5’- TTGCAGGCCGAGATTCTTCC-3’  R: 5’- ATGATTGGGCATCCTGCGAA-3’ |
| *MmSWEET15_3* | F: 5’- ACAATGGGGTTCCAATCGCT-3’  R: 5’- TTGCATGTGGAGTGGCGTAT-3’ |
| *MmBGLU47* | F: 5’- GGTGGTTGGCTAAGTCCACA-3’  R: 5’- TCAGAGCATCTTGCTGGTGG-3’ |
| *MmCEL3* | F: 5’- CTCTGTCTCCCGGACCAAAC-3’  R: 5’- AAAATGGGCAAACCGCTGAC-3’ |
| *MmSS2* | F: 5’- GAGGCGATTCCTTGGATGGT-3’  R: 5’- TTGACCGAAAACCCAACCCA-3’ |
| *MmBAM1* | F: 5’- AGATGCGGGATCATGAGCAG-3’  R: 5’- TCGTGCGCATATTCGTCGTA-3’ |
| *MmYUC10.3* | F: 5’- GAGGTTGATGGGTTGGGTGA-3’  R: 5’- TGATTGGCCAGATCGAGTGC-3’ |
| *MmNIT4* | F: 5’- TCATCCAGGCTTCCACCATC-3’  R: 5’- GCGTACGGTTACCGATTGTC-3’ |
| *MmTIR1.2* | F: 5’- AACTTTGAACGGGTGTCGGG-3’  R: 5’- AGGGTTAGGACGAATGGTGG-3’ |
| *MmARF8* | F: 5’- GAACCACCAGAGCTCGTTCA-3’  R: 5’- AAAACGAACCCAAAAGCCCG-3’ |
| *MmIPT3.1* | F: 5’- GGGTCGTGAAGCCAATGAGT-3’  R: 5’- CTCCACCGCTCTTTTCGTCA-3’ |
| *MmGLU1* | F: 5’- GAAGCACCAAAGGTGCGGTT-3’  R: 5’- CTTGACCGGTGGTTGTTTGC-3’ |
| *MmAHP5* | F: 5’- CATAGGGGCACAGCGTGTAA-3’  R: 5’- CCGGAACAAGGCTTCGAGTT-3’ |
| *MmARR6.1* | F: 5’- CCGTTGACGACAGCAACATC-3’  R: 5’- AAACCCGAACCGTCATCGAA-3’ |
| *MmKO2* | F: 5’- TGGAGGGAAAAGGGTATGCG-3’  R: 5’- CTGGGTCGTAAGCCCGATAG-3’ |
| *MmGA3OX1.1* | F: 5’-CCTCAACAGTCCGAACGTCA-3’  R: 5’- CACCGTTTGGAGCTCTAGCA-3’ |
| *MmGID1B* | F: 5’- ACCGGACCACTAGTCTCCTC-3’  R: 5’- TTGGCGGACGAATGAGTGAA-3’ |
| *MmRGL2.2* | F: 5’- CAAGAAATGGAACGGGTCGC-3’  R: 5’- CAAGGATGGCTGAATTGGCG-3’ |
